# Symbiont-derived sphingolipids modulate mucosal homeostasis and B cells in teleost fish

**DOI:** 10.1038/srep39054

**Published:** 2016-12-14

**Authors:** Ali Sepahi, Héctor Cordero, Howard Goldfine, Maria Ángeles Esteban, Irene Salinas

**Affiliations:** 1 Center for Evolutionary and Theoretical Immunology, Department of Biology, University of New Mexico, Albuquerque, NM, USA; 2 Fish Innate Immune System Group, Department of Cell Biology and Histology, Faculty of Biology, Regional Campus of International Excellence “Campus Mare Nostrum”, University of Murcia, Murcia, Spain; 3 Department of Microbiology, University of Pennsylvania Perelman School of Medicine, Philadelphia, PA 19104, USA

## Abstract

Symbiotic bacteria and mucosal immunoglobulins have co-evolved for millions of years in vertebrate animals. Symbiotic bacteria products are known to modulate different aspects of the host immune system. We recently reported that *Flectobacillus major* is a predominant species that lives in the gill and skin mucosal surfaces of rainbow trout (*Oncorhynchus mykiss*). *F. major* is known to produce sphingolipids of a unique molecular structure. Here we propose a role for *F. major* and its sphingolipids in the regulation of B cell populations in rainbow trout, as well as an essential role for sphingolipids in trout mucosal homeostasis. We found that *F. major*-specific IgT titers are confined to the gill and skin mucus, whereas *F. major*-specific IgM titers are only detected in serum. Live *F. major* cells are able to stimulate sustained IgT expression and secretion in gills. *F. major* sphingolipids modulate the growth of trout total skin and gill symbiotic bacteria. *In vivo* systemic administration of *F. major* sphingolipids changes the proportion of IgT^+^ to IgM^+^ B cells in trout HK. These results demonstrate the key role of the symbiont *F. major* and its sphingolipids in mucosal homeostasis via the modulation of mucosal and systemic Igs and B cells.

The co-existence of beneficial microorganisms and the mucosal barriers of animals is one of the most conserved and successful associations found in nature. Microorganisms are known to provide the animal host with numerous physiological benefits including metabolic, developmental and immunological ones^1^,^2^,^3^,^4^,^5^,^6^. At the same time, the animal host needs to tolerate symbionts while fighting pathogens, a complex process for the animal’s immune system^1^,^7^,^8^.

Teleost fish such as rainbow trout (*Oncorhynchus mykiss*) have numerous mucosal barriers such as the gut, skin, gills and olfactory organ that separate them from the environment. Each of these surfaces is colonized by a distinct and diverse bacterial community^1^,^9^,^10^,^11^,^12^. Although the presence of these complex microbial communities has been reported in a number of teleosts, the specific mechanisms by which the fish host benefits from this association are largely unknown.

Mucosa-associated lymphoid tissues (MALT) of teleost fish are characterized by a unique distribution of B cells compared to systemic lymphoid tissues, with 50% of all B cells being IgT^+^ B cells and 50% IgM^+^ B cells^11^,^13^,^14^,^15^. Importantly, teleost mucosal secretions contain one major immunoglobulin (Ig) isotype, IgT, specialized in mucosal immunity^11^,^13^,^14^,^15^,^16^. Compartmentalized IgT responses against pathogenic bacteria and parasites can be detected in mucosal secretion of trout whereas IgM responses are mainly systemic^11^,^13^,^14^,^15^. Additionally, rainbow trout IgT is the main Ig to coat bacterial symbionts, supporting the role of IgT in mucosal homeostasis^11^,^13^,^14^,^15^. Thus, IgT similar to IgA in mammals, is essential for the correct functioning of the teleost mucosal immune system.

A recent topographical map of the bacterial microbiome of adult rainbow trout revealed that the skin and gill bacterial communities are dominated by one species of bacteria, *Flectobacillus major*^12^. Until then, *F. major* had not been reported to be a member of the microbiome of any fish species, likely due to the lack of pyrosequencing studies from these two sites. This strong association, nevertheless, suggested that *F. major* may play a major role in the gill and skin mucosal immune system of rainbow trout.

Sphingolipids are known to perform several immune-related functions^17^,^18^,^19^. For instance, sphingolipids have antimicrobial properties and they are able to modulate immune cells by formation of secondary messengers such as ceramides and sphingosine-1 phosphate (S1P) that are involved in immune cell development, differentiation, activation and proliferation^17^. The sphingosine-1 phosphate receptor (S1P1) is mainly expressed by lymphocytes and determines their migration patterns from and into the secondary lymphoid organs and thymus^20^,^21^,^22^,^23^. Moreover, S1P/S1P1 regulate peritoneal B cell trafficking and intestinal IgA production in mice^24^,^25^.

Sphingolipids are produced by most eukaryotic cells but are rare in prokaryotes, whose membranes comprise only glycerol-based phospholipids^26^. However, a few bacterial species possess both phospholipids and sphingolipids^27^,^28^,^29^. Importantly, bacterial derived sphingolipids can have unique properties compared to those synthesized by eukaryotes^30^. Interestingly, *F. major* is known to produce large quantities of a unique type of glycosphingolipid^31^,^32^, but the biological functions of these sphingolipids have not been investigated.

Here we propose that *F. major* and *F. major*-derived sphingolipids play a key role in the modulation of trout mucosal homeostasis and B cell populations. *F. major*-specific IgT antibodies were found in the gill mucus of healthy rainbow trout whereas *F. major*-specific IgM antibodies were confined to the serum. *F. major* stimulated sustained IgT but not IgM expression in gill tissue. Sphingolipid metabolism was not only essential for *F. major* growth but also impaired the growth of other resident aerobic bacterial symbionts. Finally, we demonstrate that *F. major* sphingolipids control the distribution of IgT and IgM B cells at mucosal and mucosal sites *in vivo*. Our results show for the first time that sphingolipids produced by a bacterial symbiont are able to modulate B cells and Igs in vertebrates.

## Results

### *F. major*-specific IgT is found in trout mucus and *F. major*-specific IgM in serum

**Figure 1:**
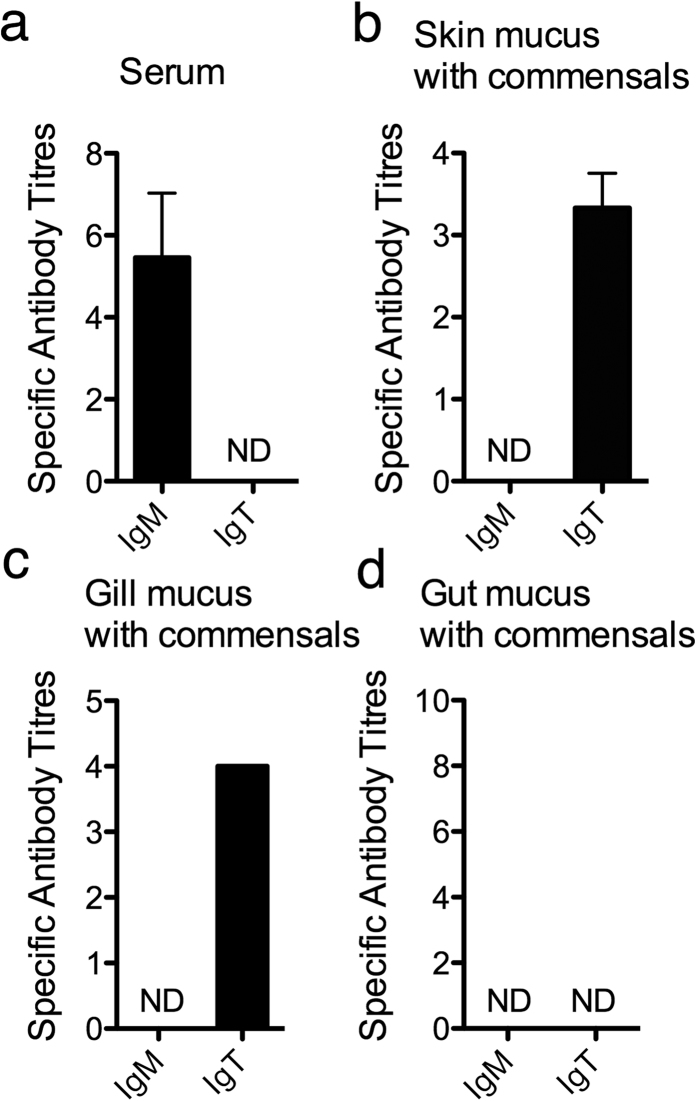
*F. major*-specific IgM and IgT can be detected in hatchery rainbow trout (*O. mykiss*). Antibody titres were measured by ELISA. Results are expressed as the mean titre of all fish that tested positive ± SEM (N = 6–10). Groups are *F. major*-specific IgM and IgT titres in rainbow trout serum (**a**) *F. major*-specific IgM and IgT titres in rainbow trout gill mucus containing bacteria (**b**) *F. major*-specific IgM and IgT titres in rainbow trout skin mucus containing bacteria (**c**) *F. major*-specific IgM and IgT titres in rainbow trout gut mucus containing bacteria (**d**).

We found specific *F. major*-IgM antibodies (titers between 1/4 and 1/6) in the serum of 36% of all analyzed specimens. However, *F. major*-specific IgT could not be detected in serum samples (Table 1, Fig. 1). In mucus, specific IgM titres were undetectable in all cases (Fig. 1). However, *F. major*-specific IgT titers were found both in gill and skin mucus but not gut mucus, with higher titers found in the gills compared to skin (Table 1, Fig. 1). Interestingly, upon symbiont removal, specific IgT could no longer be detected in skin mucus samples indicating that all the *F. major*-specific IgT in skin mucus was bound to bacteria present in the samples. In contrast, removal of bacteria from gill mucus did not eliminate the presence of specific IgT in the sample, indicating that *F. major*-specific IgT antibodies are present in free form (unbound to bacteria) in trout gill mucus (Table 1, Fig. 1). These results demonstrated the presence of Ig responses to symbiotic bacteria in mucosal and systemic compartments of teleost fish.

### *F. major* induces sustained expression of IgT but not IgM in trout gill explants

**Figure 2:**
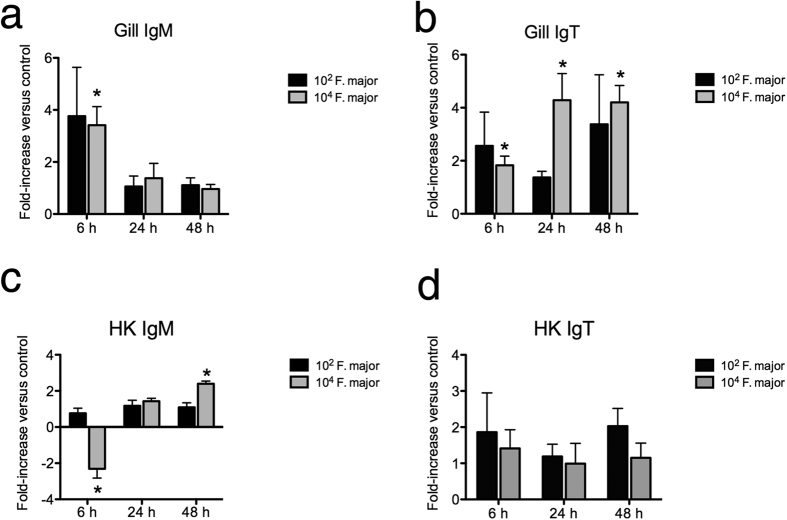
*F. major* stimulates IgM and IgT expression in gill and HK *in vitro* in a dose-dependent manner. (**a**) IgM expression in trout gill following incubation with 10^2^ cfu/ml or 10^4^ cfu/ml *F. major* for 6, 24 or 48 h. (**b**) IgT expression in trout gill following incubation with 10^2^ cfu/ml or 10^4^ cfu/ml *F. major* for 6, 24 or 48 h. (**c**) IgM expression in trout HK following incubation with 10^2^ cfu/ml or 10^4^ cfu/ml *F. major* for 6, 24 or 48 h. (**d**) IgT expression in trout HK following incubation with 10^2^ cfu/ml or 10^4^ cfu/ml *F. major* for 6, 24 or 48 h. Results are expressed as the mean fold-change compared to unstimulated control as measured by RT-qPCR (N = 5). *Denotes statistically significant changes compared to the unstimulated control (p < 0.05).

Trout gill explants incubated with 10^4^ cfu/ml of *F. major* showed significantly higher expression of IgT (between 2 and 4 fold higher than controls) at 6, 24 and 48 h whereas the lower dose (10^2^ cfu/ml) did not significantly change IgT expression. IgM expression was transiently up-regulated at 6 h with 10^4^ cfu/ml *F. major* (Fig. 2a and b) with no changes recorded at later time points or at the lower dose tested. IgT expression was not modified in head kidney leukocytes (HKLs) at any time point or dose tested. IgM expression was significantly lower (2-fold) at 6 h in the presence of 10^4^ cfu/ml *F. major* and 2-fold higher at 48 h with the same bacterial dose (Fig. 2c and d). No changes in IgM expression were detected in HKLs incubated with 10^2^ cfu/ml *F. major.* This experiment showed that the symbiont *F. major* is capable of modifying IgT and IgM transcript levels, but primarily stimulates mucosal IgT expression.

### *F. major* sphingolipids affect the growth of total trout aerobic symbionts

**Figure 3:**
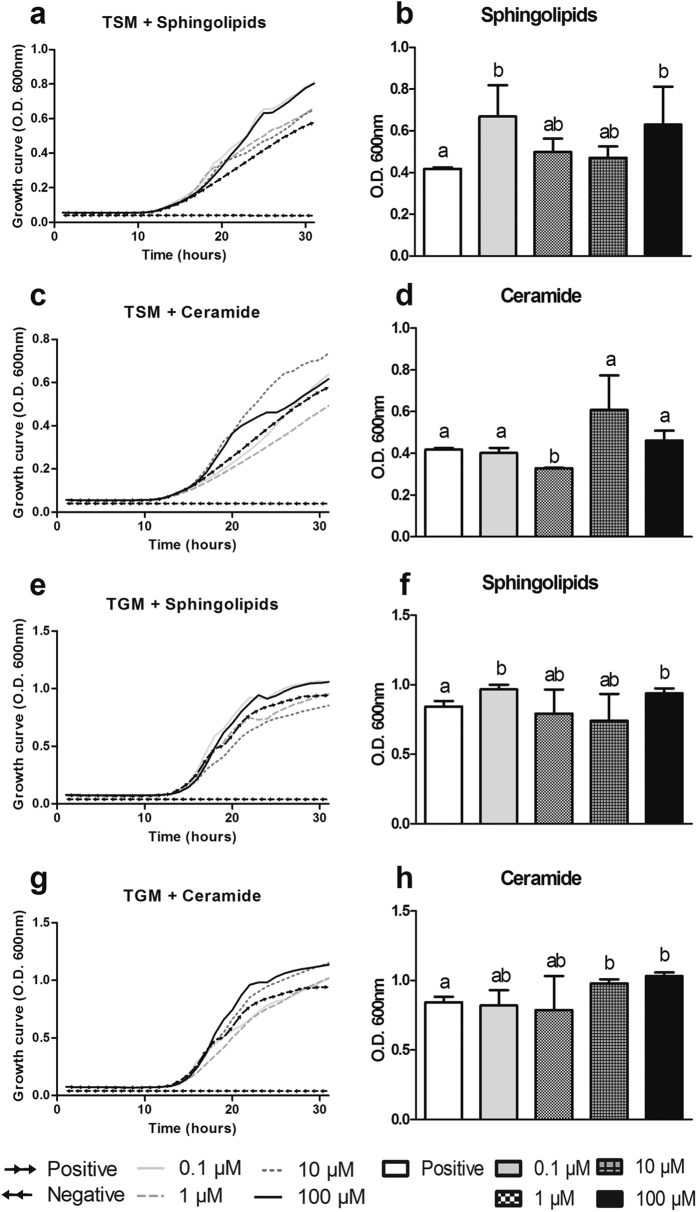
*F. major* sphingolipids modulate the growth of other trout bacterial symbionts. Effects of *F. major*-sphingolipids on trout total skin aerobic microbiota (TSM) growth over a 30 h period (**a**) or after 24 h (**b**). Effects of commercial ceramide on TSM growth over a 30 h period (**c**) or after 24 h (**d**). Effects of *F. major* sphingolipids on trout total gill aerobic microbiota (TGM) growth over a 30 h period (**e**) or after 24 h (**f**). Effects of commercial ceramide on TGM growth over a 30 h period (**g**) or after 24 h (**h**). Results are expressed as the mean and/or the mean ± SEM (N = 3). Different letters denote statistically significant differences among treatments (p < 0.05).

The growth of the total skin microbiota (TSM) of rainbow trout increased in the presence *F. major* sphingolipids at the four tested concentrations (0.1 μM, 1 μM, 10 μM and 100 μM) compared to the negative control groups (BSA only) although only the 0.1 and 100 μM doses resulted in significant growth enhancement at 24 h (Fig. 3a,b). In the case of ceramide, the substrate for the sphingolipid synthesis pathway, no significant effects on TSM growth were recorded except for a reduction in growth at 24 h in the presence of 1 μM ceramide (Fig. 3c,d). Regarding total aerobic gill microbiota (TGM) of rainbow trout, significant growth enhancement was observed in the presence of the highest (100 μM) and lowest (0.1 μM) doses of sphingolipids from *F. major* (Fig. 3e,f). Ceramide treatment, on the other hand, caused a dose-dependent growth enhancement on TGM after 24 h (Fig. 3g,h). These results indicated that *F. major*-derived sphingolipids promote TSM and TGM growth when present at low or high doses.

### *F. major* regulates the expression of genes involved in the sphingolipid and phospholipid metabolism of the host

**Figure 4:**
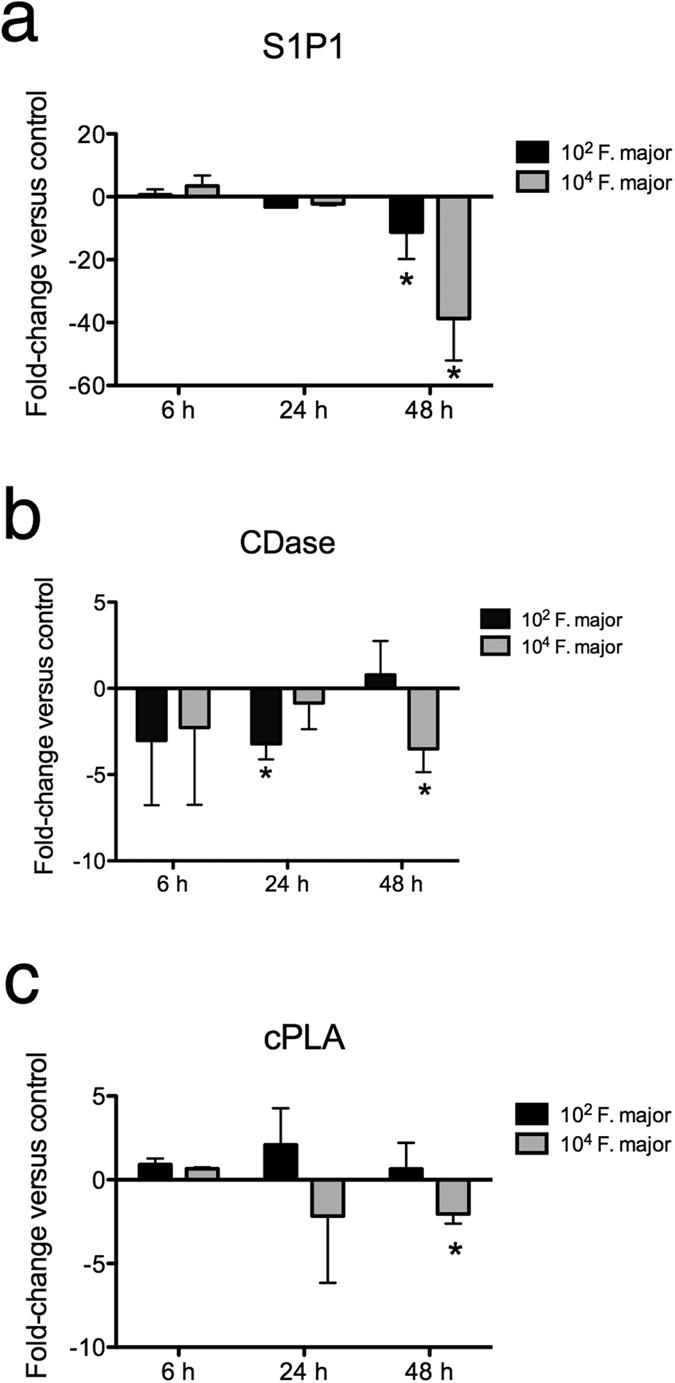
*F. major* regulates the expression of lipid metabolism genes in rainbow trout gill. Expression of (**a**) S1P1 (**b**) CDase and (**c**) cPLA in trout gill explants following incubation with 10^2^ cfu/ml or 10^4^ cfu/ml *F. major* for 6, 24 or 48 h. Results are expressed as the mean fold-change ± SEM compared to unstimulated control as measured by RT-qPCR (N = 5). *Denotes statistically significant changes compared to the unstimulated control (p < 0.05).

Since *F. major* may be a source of sphingolipids to the trout host, we sought to test whether this bacterium can modify the expression of genes involved in the sphingolipid and phospholipid metabolic pathways of the host. Incubation of gill explants with 10^4^ cfu/ml *F. major* led to significant down-regulation of S1P1, cytosolic phospholipase A2 (cPLA) and alkaline ceramidase (CDase) after 48 h, with the greatest inhibition (~40-fold) observed for S1P1 transcripts. S1P1 expression was also significantly down-regulated (~10-fold) in gill explants incubated with 10^2^ cfu/ml *F. major* for 48 h. Finally, CDase expression was significantly lower (3-fold) at 24 h in gill explants incubated with 10^2^ cfu/ml *F. major* (Fig. 4a–c). Thus, *F. major* appeared to regulate three genes involved in the host’s sphingolipid and phospholipid metabolism at mucosal sites.

### Phylogenetic analysis of salmonid S1P1

In order to gain further insights into the evolution of S1P1 in vertebrates we datamined S1P1 molecules in NCBI as well as the rainbow trout and Atlantic salmon genomes. S1P1 sequence alignment is shown in Supplementary Fig. S1. We identified two S1P1 genes in both rainbow trout and salmon that shared a high degree of amino acid sequence identity with other teleost S1P1 molecules as well as mammalian S1P1 (Supplementary Fig. S2). The Neighbour Joining tree (Supplementary Fig. S3) showed that all salmonid S1P1 form a clade that is closely related to other teleost S1P1 molecules. Teleost S1P1 clade is closely related to all tetrapod S1P1 molecules. Finally, evaluation of synteny revealed a high degree of conservation in the local genomic regions surrounding S1P1 across vertebrate species (Supplementary Fig. S4). The genes in conserved synteny included coiled-coil domain containing 76 [Source:ZFIN; Acc:ZDB-GENE-050327-19], leucine rich repeat containing 39 [Source:ZFIN; Acc:ZDB-GENE-050417-279], dihydrolipoamide branched chain transacylase E2 [Source:ZFIN; Acc:ZDB-GENE-050320-85], RNA terminal phosphate cyclase domain 1 [Source:ZFIN; Acc:ZDB-GENE-030131-9687], CDC14 cell division cycle 14 homolog A, b [Source:ZFIN; Acc:ZDB-GENE-070705-309], G protein-coupled receptor 88 [Source:HGNC Symbol; Acc:4539], vascular cell adhesion molecule 1 [Source:ZFIN; Acc:ZDB-GENE-070209-238], solute carrier family 30 (zinc transporter), member 7 [Source:ZFIN; Acc:ZDB-GENE-030131-5650], DPH5 homolog (*S. cerevisiae*) [Source:ZFIN; Acc:ZDB-GENE-041114-85] and sphingosine-1-phosphate receptor 1 [Source:ZFIN; Acc:ZDB-GENE-001228-2].

### *F. major* and *F. major* sphingolipids induce IgT but not IgM production in trout gills explants

**Figure 5:**
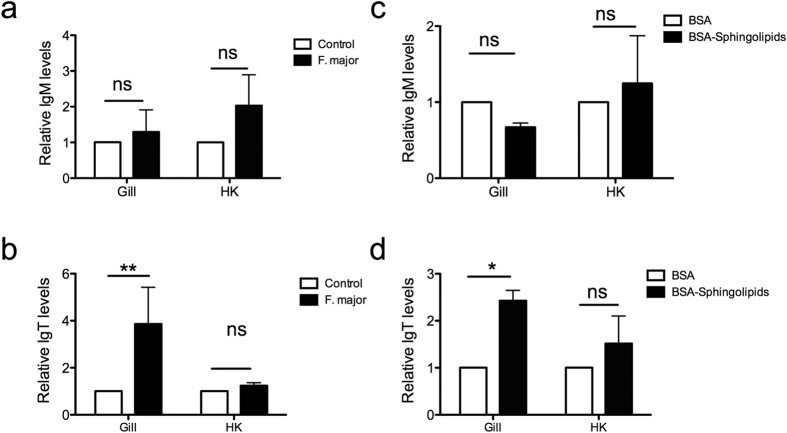
*F. major* and *F. major*-derived sphingolipids stimulate Ig production in trout gills *in vitro*. Rainbow trout gill and HK tissue explants (N = 5) were incubated with DMEM alone (control), live *F. major* cells, *F. major* sphingolipids conjugated with BSA or BSA alone. Explant supernatants were collected and total IgM (**a**,**c**) and total IgT (**b**,**d**) levels were measured by Western Blot. Results are expressed as the mean relative IgM or IgT protein levels ± SEM compared to their respective controls. Results are representative of two independent experiments. *Indicates p < 0.05, **Indicates p < 0.01.

In order to investigate if *F. major* or its sphingolipids play a role in antibody production at mucosal and systemic lymphoid tissues, we performed a number of *in vitro* experiments with live *F. major* or *F. major* sphingolipids using trout tissue explants and measure antibodies by western blot. Live *F. major* cells induced increases in IgM production in gills and HK but they were not significant (Fig. 5a and Supplementary Fig. S5). A significant increase in IgT production was detected in gill explants but not HK explants exposed to live *F. major* (Fig. 5b and Supplementary Fig. S5). Moreover, *F. major* sphingolipids did not significantly stimulate IgM production in gills or HK *in vitro* (Fig. 5c and Supplementary Fig. S5). However, *F. major* sphingolipids significantly induced IgT production in gill explants but not HK explants (Fig. 5d and Supplementary Fig. S5). These results showed that the symbiont *F. major* as well as *F. major* sphingolipids modulate IgT responses at the protein level in rainbow trout gill.

### *F. major* sphingolipids regulate the distribution of IgT^+^ B cells in trout lymphoid tissues

**Figure 6:**
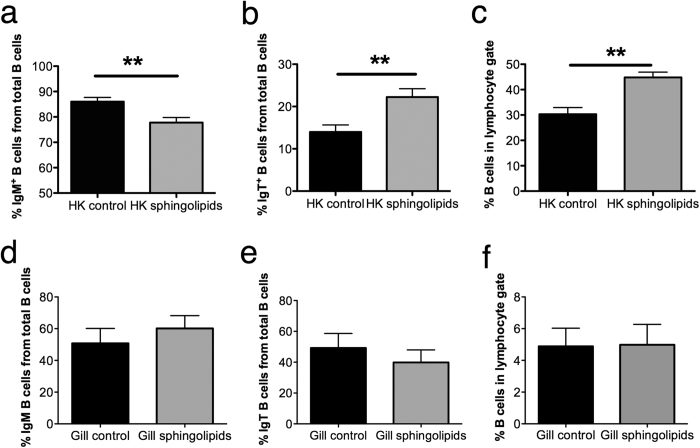
Systemic delivery of *F. major*-derived sphingolipids changes the proportions of B cell subsets and increase the total number of B cells in HK but not gills. Rainbow trout (N = 6) were injected i.v with *F. major* sphingolipids combined with BSA or BSA alone. Fish were sampled 60 h later and leukocytes isolated from the HK and gills. Percentages of IgM^+^ and IgT^+^ B cells were measured by flow cytometry. (**a**) Mean percentage of IgM^+^ cells in HK. (**b**) Mean percentage of IgT^+^ cells in HK. (**c**) Total percentage of B cells in the lymphocyte gate in HK. (**d**) Mean percentage of IgM^+^ cells in gills. (**e**) Mean percentage of IgT^+^ cells in gills. (**f**) Total percentage of B cells in the lymphocyte gate in gills. Results are expressed as the mean % of B cells ± SEM. Results are representative of four independent experiments. **Denote statistically significant differences compared to the control group (p < 0.01).

We hypothesized that if symbiont-derived sphingolipids play a role in maintaining high IgT^+^ B cells numbers at mucosal sites, then systemic delivery of these sphingolipids would result in a change of IgT/IgM ratios in systemic lymphoid tissues. Consistent with our hypothesis, we found that in BSA treated control HKLs, ~86% of all B cells were IgM^+^ and ~14% were IgT^+^. Following i.v injection of *F. major* sphingolipids the proportions of IgM^+^ B cells and IgT^+^ B cells in the HKLs were significantly changed with IgM^+^ and IgT^+^ B cells contributing to ~78% and ~22% of all B cells, respectively (Fig. 6a,b). Additionally, the proportion of all B cells within the lymphocyte gate in the HK significantly raised from ~30% in controls to ~45% in the sphingolipid treated group (Fig. 6c). As expected in the control group, gill B cells consisted of ~50% of IgM^+^ and ~50% IgT^+^ B cells. In response to the sphingolipid i.v injection, these proportions did not change significantly neither did the total number of B cells present in the gills (Fig. 6d–f). These results demonstrated that *F. major* sphingolipids, when delivered systemically, are able to increase the proportion of B cells within the HK and, specifically, to increase the proportion of IgT^+^ to IgM^+^ B cells in this organ.

## Discussion

Vertebrate mucosal surfaces have co-evolved with symbiotic microorganisms for over 500 million years. In order to co-exist with these complex microbial communities, mucosal surfaces secrete mucosal Igs that prevent microbial colonization. Symbiotic bacteria, in turn, stimulate mucosal Ig secretion^33^,^34^. Apart from Ig secretion, this intimate relationship brings vast benefits to the host including adequate development, physiology and immunity^3^,^4^,^35^,^36^.

Similar to mammalian IgA, teleost IgT is produced at mucosal sites in response to parasitic or bacterial infection, whereas systemic adaptive immune responses are characterized by specific IgM production^11^,^13^,^14^,^15^. Importantly, IgT, like IgA, keeps commensal bacteria at check through the process of immune exclusion^11^,^13^,^14^,^15^.

Among the vast diversity of microorganisms living at vertebrate mucosal epithelia, certain species appeared to have been selected through evolution due to the specific benefits that they provide to the host. We selected the symbiont *F. major* based on previous studies performed in our laboratory that revealed the high abundance of this species in the skin and gill microbiota of hatchery rainbow trout^12^. In that study, we found *F. major* as part of the microbiome of adult outbred rainbow trout and therefore different genetic background. However, whether the skin and gill microbiomes of rainbow trout from other environments is also dominated by this species is currently unknown. Here we report for the first time the presence of symbiont-specific IgT responses in mucosal secretions as well as symbiont-specific IgM responses in plasma of rainbow trout. The observed IgT and IgM antibody titres were low in all samples, suggesting that these antibodies may be natural antibodies or antibodies that are cross-reactive against a number of symbiotic species. Critically, we found *F. major*-specific titers in skin and gill mucus but not in gut mucus, indicating that Ig responses to symbionts in teleost fish, similar to mammals^37^, are tissue specific.

Additionally, we identified differences between the skin and gill IgT *F. major*-specific antibodies. Whereas in the skin all *F. major*-specific IgT was bound to bacteria, in the gills, titers were still detected after bacterial cells had been removed. This result suggests different tissue dynamics in these two sites and can be explained by a number of scenarios. It is possible that in the skin, symbiont-specific IgT production is tightly regulated by the local bacterial communities present, with no excess IgT being produced to reach the unbound, free state. An alternative explanation is that in the gills, removal of symbionts still leaves unbound IgT in the gill mucus and these unbound antibodies have a greater cross-reactivity with *F. major* than the free IgT found in the skin.

Symbiotic bacteria regulate the host physiology via the production of different metabolites^38^. Whereas sphingolipids are produced by most eukaryotic cells, prokaryotes rarely synthesize sphingolipids. However, a few examples of sphingolipid producing bacteria have been reported^29^,^30^,^32^, including the mammalian gut symbiont *Bacteroides fragilis*^27^,^30^,^39^. Symbiont-derived sphingolipids may have unique molecular structures that may confer them unique functional properties. For instance, sphingolipids from *B. fragilis* have anti-inflammatory properties and regulate iNK T cells responses in the gut of mammals^30^. Moreover, sphingolipid production is critical for the growth of *B. fragilis*^27^. Since *F. major* is a predominant commensal species of rainbow trout and previous reports had identified this species as a major producer of unique sphingolipids, we hypothesized that *F. major* sphingolipids play a major role in antibody and B cells responses at mucosal sites.

It is worth mentioning that our study was not performed with purified sphingolipids. The methodology used to extract *F. major* lipids has been shown to extract lipids other than sphingolipids^32^. These amount to approximately 11% of total polar lipids. Additionally, the preparation should also contain ~10% non-polar lipids but these should have been removed during the clean-up step. The two other polar lipids present in our preparations apart from sphingolipids are phosphatidylethanolamine and a monoglycosyldiacylglycerol^32^. We speculate that these two polar lipids are unlikely to stimulate the teleost immune system since phosphatidylethanolamine is common to teleost fish and monoglycosyldiacylglycerol is common to many bacteria, although further research is needed in this area. We found that *F. major* sphingolipids were able to modulate the growth of other aerobic symbionts isolated from trout skin and gills. The responses were not always dose-dependent, since intermediate concentrations (1 and 10 μM) of the sphingolipids did not stimulate bacterial growth but low and high concentrations did. Thus, these results may suggest that different bacterial species have different capabilities to metabolize lipids; some bacteria may sense the concentration gradients of these sphingolipids as growth factors whereas others may suffer from their antimicrobial effects and therefore the combined effects on the overall growth of the microbiota may be masked under specific sphingolipid concentrations. Additionally, the total trout aerobic bacteria community measured in this study is biased by the culture conditions and does not capture the entire breadth of modulatory growth effects that this product may have in non-culturable bacterial species. This capability from a bacterial symbiont to modulate the microbiota through sphingolipid pathway is an interesting finding that deserves further investigation.

*F. major* was able to stimulate IgT gene expression in trout gills but not HK *in vitro* whilst IgM stimulation was modest. In support, *F. major* and *F. major* sphingolipids induced secretion of IgT but not IgM antibodies in gills but not HK *in vitro,* suggesting that they can induce differentiation of B cells into antibody secreting cells. These results also indicate the existence of unique differentiation programs of B cells into antibody secreting cells in gills compared to HK. Finally, IgT but not IgM secretion was stimulated highlighting the intimate co-evolution between symbionts and mucosal antibodies of vertebrates.

Because we found that *F. major* cells increase the expression of antibody transcripts *in vitro*, we hypothesized that sphingolipids produced by this symbiont play a role in the distribution of trout B cell populations. A number of studies have demonstrated that at mucosal sites of trout such as the gut, skin, gill and nose, the ratio of IgT to IgM B cells is 1:1^11^,^13^,^14^,^15^. This is in sharp contrast to the preponderance of IgM B cells in systemic lymphoid tissues such as the HK, the spleen and the blood^14^,^16^. However, the mechanisms contributing to this unique distribution of B cells at trout mucosal lymphoid tissues is unknown. In mammals, host-derived sphingolipids are known to control lymphocyte trafficking in and out of lymphoid tissues^20^,^21^,^23^,^40^,^41^,^42^,^43^,^44^,^45^. Moreover, host-derived sphingolipids were shown to determine IgA B cell trafficking in mice^23^,^24^. Since under natural conditions, trout skin and gill B cells may be exposed to *F. major* sphingolipids, we hypothesized that these sphingolipids may be responsible for the maintenance of IgT^+^ B cells at these two mucosal sites. In order to test this, we delivered sphingolipids systemically, expecting to shift the proportions of B cells in the HK towards a more “mucosal-like” distribution. We found that symbiont-derived sphingolipids increase the proportion of B cells in the HK. The latter may be achieved by local production of B cells or recruitment. Importantly, *F. major* sphingolipids shifted the proportion of IgT^+^ to IgM^+^ B cells, which again shows that this symbiont product preferentially recruits or stimulates production of IgT^+^ B cells over IgM^+^ cells. These data are not in line with our gene expression results *in vitro* performed with the whole *F. major* bacterium, where no significant increase in IgT expression was recorded in HK. Although both experiments are not directly comparable, this disparity may imply that effects of sphingolipids on HK B cell populations require the intact HK microenvironment for B cells to proliferate or the influx of B cells from other lymphoid organs into the HK. Both requirements are met in the *in vivo* injection experiments but not in the *in vitro* ones.

Our *in vivo* results using *F. major* sphingolipids suggested that trout B cells may have receptors that bind these sphingolipids. In mammals, S1P1 receptor binding to its ligand S1P leads to the internalization of this complex^46^. The reduced S1P1 expression on the surface of lymphocytes results, in turn, in the sequestration of lymphocytes within lymphoid tissues therefore preventing them from entering in circulation^20^,^21^,^23^,^40^,^41^,^42^,^43^,^44^,^45^. Additionally, S1P has been shown to control intestinal IgA production^24^,^25^. In teleosts, S1P1 controls venous vascular integrity and development^47^ but its functional role in immune cells is so far unknown. We found that *F. major* is able to reduce the expression of S1P1 in the gill of rainbow trout. This could potentially lead to the sequestration of IgT B cells produced in the HK and circulating through the gills. Future studies should address the particular S1P1 expression patterns in IgT^+^ and IgM^+^ B cells and whether mucosal versus systemic B cells display different levels of expression of this receptor.

In conclusion, the present study demonstrates the intimate relationship between the symbiont *F. major* and the immune system of rainbow trout. Our results support the idea of an ancient co-existence between symbiotic bacteria and mucosal Igs and B cells. Moreover, sensing of symbiont-derived sphingolipids by trout B cells may be a novel mechanism of symbiont regulation of the host immune system. Fish symbiont sphingolipids could have beneficial applications for the aquaculture industry due to their IgT modulatory properties.

## Materials and Methods

### Animals, serum and mucus sample collection

Healthy adult triploid rainbow trout (*O. mykiss*) with a mean body weight of 250 ± 20 g were obtained from Lisboa Springs Fish Hatchery (Pecos, New Mexico, USA). Fish were anesthetized with MS-222 and bled from the caudal vein with a heparinized 3 mL syringe. Plasma samples were collected and stored at −80 °C until use. Total gill, gut and skin mucus were collected with a sterile cell scraper as explained elsewhere^13^,^14^. Half of the sample was used directly for antibody titer measurement (containing all Igs, bound to bacteria and not bound to bacteria) whereas the other half was subject to a series of centrifugation steps as explained elsewhere^13^. At the end of this procedure, the supernatants were filtered through a 0.2 μm filter in order to detect antibodies unbound to bacteria. The pellets were used for the growth assays described below. All animal studies were reviewed and approved by the Institutional Animal Care and Use Committee (IACUC) at the University of New Mexico, protocol number 16-200384-MC. All methods were performed in accordance with the relevant guidelines and regulations.

### Bacteria culture

*F. major* was grown in the specific culture medium ATCC^®^ 29496TM at 25 °C for 30 h in 956 *Microcyclus* medium as per manufacturer’s instructions, and adjusted to the desirable concentration in each case.

### Sphingolipid extraction and purification

Five hundred ml of an *F. major* culture grown for 30 hours were centrifuged at 4000 g for 15 min. Supernatants were discarded and pellets were washed once in PBS. Pellets were left to dry in a sterile tissue culture hood for 4 h and then frozen at −20 °C until lipid extraction. Lipids were extracted as explained elsewhere^31^ and then cleaned-up using a G25 Sephadex column as explained elsewhere^48^. In order to avoid toxicity to cells when used *in vitro*, purified sphingolipids and ceramide (a simple glycosphingolipid that serves as the substrate for the sphingolipid synthesis pathway), were combined with a 2 mM bovine serum albumin solution in Dulbecco’s Modified Eagle Medium (DMEM, Life Technologies).

### *In vitro* exposure of rainbow trout gill and HK to *F. major* and *F. major* sphingolipids

After serum and mucus collection, gill tissue was excised with sterile scissors and placed in a Petri dish. Gills were rinsed until all the blood was removed by injecting sterile cold PBS into the gill arch with a 1 ml syringe. Blood-free gill samples were excised into 0.5 cm wide pieces and placed in flat bottom 24-well plates. Trout HK and HKLs were obtained as explained elsewhere^11^ and seeded onto flat-bottom 24-well plates at 10^6^ cells/well. Both gills and HK explants (N = 5) were cultured in 1 ml DMEM supplemented with 10% fetal bovine serum (Sigma-Aldrich) and 1% Penicillin-Streptomycin (Gibco). For gene expression studies, gill explants and single cell HKLs suspensions were incubated for 6, 24, and 48 h with 10^2^ or 10^4^ *F. major* cfu/ml. Wells without bacteria were used as negative control. At each time point, samples were collected and placed in 1 ml TRIzol (Life Technologies) for RNA extraction according to the manufacturer’s instructions. Synthesis of cDNA was performed using 2 μg of total RNA, which was first denatured (65 °C, 5 min) in the presence of 1 μl of oligo-dT (Life Technologies), 1 μl dNTPs (10 mM each, Promega). Next, samples containing 1 μl Superscript III enzyme reverse transcriptase (Life Technologies) with 5 μl of 5x first strand buffer (Life Technologies), 1 μl of DTT 0.1 M (Life Technologies) and RNA/DNA free molecular water (Sigma-Aldrich) in a final volume of 25 μl were incubated at 55 °C for 1 h followed by 15 min at 70 °C for later qPCR analysis.

For IgT and IgM detection in supernatants, gill explants and HKLs (N = 5) were cultured for 5 days in 1 ml Dulbecco’s Modified Eagle Medium (DMEM, Life Technologies) supplemented with 10% fetal bovine serum (Sigma-Aldrich), 1% Penicillin-Streptomycin (Gibco) and 0.5 μg/ml fungizone (Gibco). Explants were incubated for 5 days with only medium (DMEM control), live *F. major* (10^4^ cfu/ml), 30 μM *F. major* sphingolipids conjugated with 2 mM BSA in DMEM; 30 μM C2-ceramide (Enzo Life Sciences) conjugated with 2 mM BSA in DMEM or 2 mM BSA alone in DMEM (BSA control). Supernatants were stored at −20 °C until use.

### Effects of *F. major* sphingolipids and ceramide on the growth of total gill and skin aerobic bacteria

Total microbiota from skin and gut mucus (N = 3) were collected as explained elsewhere^12^, washed twice in PBS and adjusted to an optical density of 0.01 in tryptic soy broth (TSB, Sigma-Aldrich). Samples were incubated with 0.1, 1, 10 and 100 μM of sphingolipids from *F. major* or commercial ceramide for 30 h and the optical density at 600 nm was measured in a plate reader (Synergy H1). Wells containing TSB only and TSB with lipids or ceramide without bacteria were used as negative controls.

### ELISA

*F. major*-specific IgM and IgT titres were measured in serum, gill, gut and skin mucus from healthy control hatchery trout (N = 10–12) using an enzyme-linked immunosorbent assay (ELISA). Mucus samples were used in whole or as bacteria-free mucus after all symbionts were removed as explained elsewhere^14^. Flat-bottomed 96-well plates were coated overnight at 4 °C with 100 μl of a *F. major* culture adjusted at 10^7^ cfu/ml in PBS. The plates were rinsed once with PBS before blocking for 2 h at room temperature with blocking solution containing 8% non-fat dry milk (LabScientific) in PBS containing 0.05% Tween 20 (PBS-T). After rinsing with PBS-T containing 10 mM EDTA (pH 7.2), the plates were then incubated for 90 min with 100 μl of diluted serum or mucus samples in PBS with 10 mM EDTA. After washing three times, 100 μl of either mouse anti-trout IgM antibody or rabbit anti-trout IgT were added to each well (1/500 in PBS-T) or their respective isotype controls (mouse IgG1 or rabbit prebleed). After washing, wells were incubated for 45 min with the corresponding secondary antibodies (HRP-conjugated donkey anti-mouse IgG or HRP-conjugated donkey anti-rabbit IgG, both at 1/1000 in PBS-T, Jackson Immunoresearch). After four washes in PBS, the plates were developed using 100 μl of a 0.42 mM solution of 3,3′,5,5′-tetramethylbenzidine hydrochloride (TMB, Sigma-Aldrich) prepared in water containing 0.01% H_2_O_2_. The reaction was stopped after 2–15 min by adding 50 μl of 2 M H_2_SO_4_. The plates were read at 450 nm in a plate reader (Synergy H1). Samples without bacteria and without serum/mucus were also used as negative controls. Positive titers were determined by subtracting the absorbance detected in the isotype controls from the absorbance in the sample wells.

### Western blotting

Western blotting was performed as explained elsewhere^11^. Briefly, 10 μl of each explant supernatant were mixed with 10 μl of Laemmli buffer (Bio-Rad) under non-reducing conditions. Samples were boiled for 3 min at 97 °C and resolved on 4–15% SDS-PAGE gels (Bio-Rad). Gels were run for 50 min at 120 V and transferred onto PVDF membranes (Amersham). Membranes were blocked in PBS-T containing 5% non-fat milk overnight at 4 °C. Membranes were incubated with rabbit anti-trout IgT (1:1000) for 90 min, washed three times in PBS-T and then incubated for 60 min with HRP-conjugated donkey anti-rabbit IgG (1:2500). Detection was performed using ECL Western Blotting Substrate (Pierce). Membranes were stripped for 20 min in stripping buffer (0.1 M Glycine, 0.02% NaN_3_, pH = 2.5) and reprobed with mouse anti-trout IgM for 90 min followed by HRP-conjugated donkey anti-mouse IgG (1:2500) for 60 min. After washing, membranes were developed as explained before. Immunoblots were scanned using a ChemiDoc XRS+ System (Bio-Rad) and band densitometry was analysed with Image Lab Software (Bio-Rad).

### RT-qPCR

cDNA synthesis was carried out using 1 μl Superscript III enzyme reverse transcriptase (Invitrogen) in the presence of 5 μl of 5x first strand buffer, 1 μl 0.1 M DTT, made up to a final volume of 25 μl with water, and incubated at 55 °C for 1 h. The resultant cDNA was stored at −20 °C. The expression of IgM, IgT, cytosolic phospholipase A2 (cPLA), ceramidase (CDase) and S1P1 was measured by RT-qPCR using specific primers (Table 2). IgM and IgT primers were designed to amplify the CH regions of the antibody molecules and therefore amplification of both functionally and non-functionally rearranged Igs was performed. The qPCR was performed using 3 μl of a diluted cDNA template as described elsewhere^49^. The relative expression level of the genes was determined using the Pfaffl method^50^.

### Sequence analysis of salmonid S1P1

Salmonid S1P1 sequences were identified by data mining in NCBI as well as the rainbow trout and Atlantic salmon genomes. Available mammalian S1P1 sequences were blasted to identify teleost S1P1 molecules. We identified zebrafish (*Danio rerio*) S1P1, nile tilapia (*Oreochromis niloticus*) S1P1 and Atlantic salmon (*Salmo salar*) S1P1. The salmon sequence was used in further searches conducted in (http://salmobase.org/) that identified a second molecule in this species names (CIGSSA_084099.t1) that was named salmon S1P1-like. The zebrafish S1P1 sequence was used in the rainbow trout genome browser Genoscope (http://www.genoscope.cns.fr/blat-server/cgi-bin/trout) using default parameters in order to find rainbow trout S1P1 sequences. In that way, we identified 4 scaffolds (6065, 406, 1988 and 2747) in the trout genome. Blast searches of each scaffold revealed that only scaffold 6065 corresponded with S1P1 whereas the other scaffolds contained S1P2 and S1P3 molecules. BlastX of scaffold 6065 identified two rainbow trout unnamed protein products, one with 100% identity with the 6065 scaffold (CDQ90261.1) and one with 97% identity (CDQ69631.1) with the 6065 scaffold. CDQ90261.1 was identical to the translated protein sequence obtained from scaffold 6065. Sequence alignments were performed in CLUSTALW and a phylogenetic Neighbor-Joining tree (10,000 bootstrap) was constructed in MEGA6. Amino acid sequence identity and similarity were determined using MatGAT. Synteny analysis was performed in Genomicus version 01.01 (http://www.genomicus.biologie.ens.fr/genomicus-trout-01.01/cgi-bin/search.pl).

### *In vivo* administration of *F. major* sphingolipids

Rainbow trout (N = 6) received 50 μl of DMEM containing 1.4 μg of *F. major* sphingolipids combined with 2 nM BSA by intravenous (i.v) injection. The negative control group received the same volume of DMEM containing BSA only. Trout were sampled 60 h post-injection. The gill and HK of each fish were collected and leukocytes isolated as explained elsewhere^15^.

### Flow cytometry

One hundred thousand gill leukocytes or HKLs from each fish were stained with mouse anti-trout IgT and mouse anti-trout IgM as described elsewhere^14^. For secondary antibodies, a FITC-conjugated rat anti-mouse IgG2b and a Dylight 649-conjugated anti mouse IgG1 (both from Biolegend) were used. A total of 20,000 events from the lymphocyte gates were collected in an Attune Flow Cytometer (Life Technologies) and analyzed in the Attune analysis software. The total number of B cells was calculated by adding the percentage of IgM^+^ and IgT^+^ cells from the lymphocyte gate in each sample.

### Statistical analysis

Results are expressed as the mean ± standard error (SE). Data analysis was performed in GraphPad Prism version 5.0. Results were analyzed by unpaired t-test, paired t-test (for IgT and IgM quantification by western blot) or ANOVA followed by post-hoc Tukey test according to each dataset to identify statistically significant differences among groups. Statistically significant differences were considered when p < 0.05, which were denoted with asterisks or different letters according to each dataset.

## Additional Information

**How to cite this article**: Sepahi, A. *et al*. Symbiont-derived sphingolipids modulate mucosal homeostasis and B cells in teleost fish. *Sci. Rep.* **6**, 39054; doi: 10.1038/srep39054 (2016).

**Publisher's note:** Springer Nature remains neutral with regard to jurisdictional claims in published maps and institutional affiliations.

## Supplementary Material

Supplementary Information

## Figures and Tables

**Table 1:** Detection of specific immunoglobulins against *Flectobacillus major* in serum and mucus of rainbow trout (*Oncorhynchus mykiss*).

Specific Ig	Sample	Positive/Total
IgM	Serum	**4/11**
	Skin mucus with bacteria	0/6
	Skin mucus without bacteria	0/6
	Gill mucus with bacteria	0/6
	Gill mucus without bacteria	0/6
	Gut mucus with bacteria	0/6
	Gill mucus without bacteria	0/6
IgT	Serum	0/6
	Skin mucus with bacteria	**6/6**
	Skin mucus without bacteria	0/6
	Gill mucus with bacteria	**6/6**
	Gill mucus without bacteria	**6/6**
	Gut mucus with bacteria	0/6
	Gut mucus without bacteria	0/6

**Table 2:** Primers used for RT-qPCR study.

Gene symbol	Accession number	Sequence (5′ → 3′)
*Ef1a*	AF498320	F: CAACGATATCCGTCGTGGCA
		R: ACAGCGAAACGACCAAGAGG
*S1P1*	BX863528	F: AAGGGAGACCGTCGTATCCT
		R: CACACACACTTGCACACTGC
*CDase*	CX034530	F: GGTTGGCATTGGATTCACTT
		R: TTGGGCAGGTATCTTTTTGG
*cPLA*	FP321558	F: GCAGTGCCTTCTCCATTCTC
		R: CCCAGGATGTGTTGAGGTTT
*IgM*	OMU04616	F: AAGAAAGCCTACAAGAGGGAGA
		R: CGTCAACAAGCCAAGCCACTA
*IgT*	AY870264	F: CAGACAACAGCACCTCACCTA
		R: GAGTCAATAAGAAGACACAACGA
