# Supplementary material for: Symbiont-derived sphingolipids modulate mucosal homeostasis and B cells in teleost fish
Source: Sci Rep. 2016 Dec 14;6:39054. doi: 10.1038/srep39054 (PMC5155239; doi:10.1038/srep39054)

Symbiont-derived sphingolipids modulate mucosal homeostasis and B cells in teleost fish

Ali Sepahi[1 ‡], Héctor Cordero[1,2 ‡], Howard Goldfine[3], Maria Ángeles Esteban[2], Irene Salinas[1]*

Supplementary Fig. S1: Amino acid sequence alignment of vertebrate S1P1 performed in CLUSTALW. Accession numbers used were: Hs S1P1 (AAH18650.1 Sphingosine-1-phosphate receptor 1 [Homo sapiens]); Hs S1P2 (AAH69598.1 Sphingosine-1-phosphate receptor 2 [Homo sapiens]); Mm S1P1 (AAH51023.1 Sphingosine-1-phosphate receptor 1 [Mus musculus]); Rn S1P1 (NP_058997.1 Sphingosine 1-phosphate receptor 1 [Rattus norvegicus]); Gg S1P1 (XP_422305.3 PREDICTED: sphingosine 1-phosphate receptor 1 [Gallus gallus]); Xt S1P1 (NP_001072893.1 sphingosine 1-phosphate receptor 1 [Xenopus tropicalis]); On S1P1 (XP_005475711.1 PREDICTED: sphingosine 1-phosphate receptor 1 [Oreochromis niloticus]); Dr S1P1 (AAG45430.1 sphingosine 1-phosphate receptor [Danio rerio]); Ss S1P1 (XP_014071480.1 PREDICTED: sphingosine 1-phosphate receptor 1 [Salmo salar]); Ss S1P1-like (CIGSSA_084099.t1); Om S1P1 (CDQ90261.1 and Scaffold 6065) and Om S1P1 (CDQ69631.1).

```
Om_S1P1_CDQ69631.1                  ----------------MGDSMYSDLIARHYNFTGKLRKVEQDS-RLKADS
CIGSSA_084099.t1                    ----------------MGDSMYSDLIARHYNFTGKLRKVEQDS-RLKADS
Om_S1P1_CDQ90261.1_Scaffold_60      ----------------MGDSMYSDLIARHYNFTGKLRKVEQDS-RLKADS
Ssalar_S1P1                         ----------------MGDSMYSDLIARHYNFTGKLRKVEQDS-RLKADS
Drerio_S1P1                         ---------------------MDDLIARHYNFTGKFRKVHKDP-GLKADS
Oniloticus_S1P1                     -------------MEAMAEPSYSDLIAKHYNYTGKFRKTEQDS-GLKADS
Mmusculus_S1P1                      -MVSTSIPEVKALRSSVSDYGNYDIIVRHYNYTGKLNIGAEKDHGIKLTS
Rnorvegicus_S1P1                    MVSSTSIPVVKALRSQVSDYGNYDIIVRHYNYTGKLNIGVEKDHGIKLTS
Hsapiens_S1P1                       -MGPTSVPLVKAHRSSVSDYVNYDIIVRHYNYTGKLNISADKENSIKLTS
Ggallus_S1P1                        --MSSGTTAPVRVVSSLTNTDVNYVIKEHYNYTGKLNENADSG--IKVTS
Xtropicalis_S1P1                    -------MTPTSATQRRNEYYDHEIIIEHYNYTGKYKG--NLSTDIKPTS
                                                            :* .***:*** .   .    :*  *

Om_S1P1_CDQ69631.1                  VVFIIVCCFIILENVLVLLTIWRTKKFHKPMYYFIGNLALSDLLAGVVYT
CIGSSA_084099.t1                    VVFIIVCCFIILENVLVLLTIWRTKKFHKPMYYFIGNLALSDLLAGVVYT
Om_S1P1_CDQ90261.1_Scaffold_60      VVFIIVCCFIILENVLVLLTIWRTKKFHKPMYYFIGNLALSDLLAGVVYT
Ssalar_S1P1                         VVFIIVCCFIILENVLVLLTIWRTKKFHKPMYYFIGNLALSDLLAGVVYT
Drerio_S1P1                         VVFIIVCCFIILENVLVLLTIWRTKKFHKPMYYFIGNLALSDLLAGVVYT
Oniloticus_S1P1                     VIFIIVCCFIILENILVLTTIWRTKKFHKPMYYFIGNLALSDLLAGVVYT
Mmusculus_S1P1                      VVFILICCFIILENIFVLLTIWKTKKFHRPMYYFIGNLALSDLLAGVAYT
Rnorvegicus_S1P1                    VVFILICCLIILENIFVLLTIWKTKKFHRPMYYFIGNLALSDLLAGVAYT
Hsapiens_S1P1                       VVFILICCFIILENIFVLLTIWKTKKFHRPMYYFIGNLALSDLLAGVAYT
Ggallus_S1P1                        VVFIIICCFIILENIFVLLTIWKTKKFHRPMYYFIGNLALSDLLAGVAYT
Xtropicalis_S1P1                    IIFIIICCFIVLENILVLLTIWRTKKFHRPMYYFIGNLALSDLLAGTAYT
                                    ::**::**:*:***::** ***:*****:*****************..**

Om_S1P1_CDQ69631.1                  ANILLSGANTYKLTPTQWFFREGSMFVALAASVFSLLAIAIERHLTMLKM
CIGSSA_084099.t1                    ANILLSGANTYKLTPTQWFFREGSMFVALAASVFSLLAIAIERHLTMLKM
Om_S1P1_CDQ90261.1_Scaffold_60      ANILLSGANTYKLTPTQWFFREGSMFVALAASVFSLLAIAIERHLTMLKM
Ssalar_S1P1                         ANILLSGANTYKLTPTQWFFREGSMFVALAASVFSLLAIAIERHLTMLKM
Drerio_S1P1                         ANILLSGANTYKLTPTQWFFREGSMFVALAASVFSLLAIAIERHLTMLKM
Oniloticus_S1P1                     ANILLSGANTYKLTPTQWFFREGSMFVALAASVFSLLAIAIERHLTMLKM
Mmusculus_S1P1                      ANLLLSGATTYKLTPAQWFLREGSMFVALSASVFSLLAIAIERYITMLKM
Rnorvegicus_S1P1                    ANLLLSGATTYKLTPAQWFLREGSMFVALSASVFSLLAIAIERYITMLKM
Hsapiens_S1P1                       ANLLLSGATTYKLTPAQWFLREGSMFVALSASVFSLLAIAIERYITMLKM
```

```
Ggallus_S1P1                        ANLLLSGHKTYSLTPSQWFVREGSMFVALSASVFSLLAIAIERYITMLKM
Xtropicalis_S1P1                    ANILLSGPHTYKLTPVEWLIREGSMFVALSASVFSLVAIAIERYITMLKM
                                    **:****  **.*** :*:.*********:******:******::*****

Om_S1P1_CDQ69631.1                  KLHNNGNTCRVFMLISTVWLIAAILGGLPIMGWNCIQSMPSCSTVLPLYH
CIGSSA_084099.t1                    KLHNNGNTCRVFMLISTVWLIAAILGGLPIMGWNCIQSMPSCSTVLPLYH
Om_S1P1_CDQ90261.1_Scaffold_60      KLHNNGNTCRVFMLISTVWLIAAILGGLPIMGWNCIQSMPSCSTVLPLYH
Ssalar_S1P1                         KLHNNGNTCRVFMLISTVWLIAAILGGLPIMGWNCIQSMPSCSTVLPLYH
Drerio_S1P1                         KLHNNGKTCRVFMLISTVWFIAAILGGLPVMGWNCIDSINNCSTVLPLYH
Oniloticus_S1P1                     KLHNNGNTFRVFLLISTVWMIAAVLGGLPVMGWNCIQSMTQCSTVLPLYH
Mmusculus_S1P1                      KLHNGSNSSRSFLLISACWVISLILGGLPIMGWNCISSLSSCSTVLPLYH
Rnorvegicus_S1P1                    KLHNGSNSSRSFLLISACWVISLILGGLPIMGWNCISSLSSCSTVLPLYH
Hsapiens_S1P1                       KLHNGSNNFRLFLLISACWVISLILGGLPIMGWNCISALSSCSTVLPLYH
Ggallus_S1P1                        KLHNGSNSFRSFLLISACWVISVILGGLPIMGWNCISLLSNCSTVLPLYH
Xtropicalis_S1P1                    KLHNGSKSSRSFLLISGCWILSLFLGGLPIMGWNCIKQISACSTVLPLYH
                                    ****..:. * *:***  *.:: .*****:******. :  *********

Om_S1P1_CDQ69631.1                  KTYILFCTTVFSVILMAIVVLYARIYALVRTRSRKMVFRKVSNGRGGGSA
CIGSSA_084099.t1                    KTYILFCTTVFSVILMAIVVLYARIYALVRTRSRKMVFRKVSNGRGGGSA
Om_S1P1_CDQ90261.1_Scaffold_60      KTYILFCTTVFSVILMAIVVLYARIYALVRTRSRKLVFRKVSNGRGGGSA
Ssalar_S1P1                         KTYILFCTTVFSVILMAIVVLYARIYALVRTRSRKLVFRKVSNGRGGGSA
Drerio_S1P1                         KAYILFCTTVFSVILMAIVILYARIYALVRTRSRKLVFRKVANGRG----
Oniloticus_S1P1                     KAYILFCTTVFSIILMAIVVLYARIYALVRTRSRKLVFRKVSNGRSNASA
Mmusculus_S1P1                      KHYILFCTTVFTLLLLSIVILYCRIYSLVRTRSRRLTFRKN------ISK
Rnorvegicus_S1P1                    KHYILFCTTVFTLLLLSIVILYCRIYSLVRTRSRRLTFRKN------ISK
Hsapiens_S1P1                       KHYILFCTTVFTLLLLSIVILYCRIYSLVRTRSRRLTFRKN------ISK
Ggallus_S1P1                        KHYILFCTTVFTGLLLSIVVLYCRIYSMVRTRSRRLTFRKN------ITK
Xtropicalis_S1P1                    KHYILFCTTIFCALLMAIVILYARIYFLVRTRSRSLTFKRN------LAR
                                    * *******:*  :*::**:**.*** :****** :.*::

Om_S1P1_CDQ69631.1                  SSKSSEKSMALLKTVIIVLSCFIACWAPLFILLLLDVACNIRMCPILYKA
CIGSSA_084099.t1                    SSKSSEKSMALLKTVIIVLSCFIACWAPLFILLLLDVACDIRMCPILYKA
Om_S1P1_CDQ90261.1_Scaffold_60      SSKSSEKSMALLKTVIIVLSCFIACWAPLFILLLLDVACDIRMCAILYKA
Ssalar_S1P1                         SSKSSEKSMALLKTVIIVLSCFIACWAPLFILLLLDVACDIRMCAILYKA
Drerio_S1P1                         SNKSSEKSMALLKTVIIVLSCFIACWAPLFILLLLDVACQTLTCSILYKA
Oniloticus_S1P1                     NSKSSEKSMALLKTVIIVLSCFIACWAPLFILLLLDVACETLSCPILYKA
Mmusculus_S1P1                      ASRSSEKSLALLKTVIIVLSVFIACWAPLFILLLLDVGCKAKTCDILYKA
Rnorvegicus_S1P1                    ASRSSEKSLALLKTVIIVLSVFIACWAPLFILLLLDVGCKAKTCDILYKA
Hsapiens_S1P1                       ASRSSEKSLALLKTVIIVLSVFIACWAPLFILLLLDVGCKVKTCDILFRA
Ggallus_S1P1                        ATRSSEKSLALLKTVIIVLSAFIACWAPLFILLLLDVGCRVKTCPILYKA
Xtropicalis_S1P1                    PSRSSEKSMALLKTVIIVLSVFILCWSPLFIFLLLDFGCKVKTCPVLFKA
                                     .:*****:*********** ** **:****:****..*    * :*::*

Om_S1P1_CDQ69631.1                  EWFLALAVLNSAMNPLIYTLTSNEMRRAFLKTLLCCSVCTQSS-GKFSKP
CIGSSA_084099.t1                    EWFLALAVLNSAMNPLIYTLTSNEMRRAFLKTLLCCSICTRPS-GKFSQP
Om_S1P1_CDQ90261.1_Scaffold_60      EWFLALAVLNSAMNPLIYTLTSNEMRRAFLKTLMCCSVCTRPS-GKFSRP
Ssalar_S1P1                         EWFLALAVLNSAMNPLIYTLTSNEMRRAFLKTLMCCCVCTRPS-GKFSRP
Drerio_S1P1                         EWFLALAVLNSAMNPLIYTLTSNEMRRAFIKMLNCG-VCVQPS-GKFSRP
Oniloticus_S1P1                     EWFLALAVLNSAMNPLIYTLTSNEMRRAFLKTLLCCTAFIRPR-TKLTGP
Mmusculus_S1P1                      EYFLVLAVLNSGTNPIIYTLTNKEMRRAFIRIVSCCKCPNGDSAGKFKRP
Rnorvegicus_S1P1                    EYFLVLAVLNSGTNPIIYTLTNKEMRRAFIRIISCCKCPNGDSAGKFKRP
```

```
Hsapiens_S1P1                       EYFLVLAVLNSGTNPIIYTLTNKEMRRAFIRIMSCCKCPSGDSAGKFKRP
Ggallus_S1P1                        EYFLVLAVLNSATNPIIYTLTNKEMRRAFIKILCCCKCPPTDSGTKFKRP
Xtropicalis_S1P1                    EYFLSLAVLNSATNPIIYTLTNREMRRAFLKMACCSHCSIFGSSSKVKRP
                                    *:** ******. **:*****..******::   *          *.. *

Om_S1P1_CDQ69631.1                  IIG-AEFSRSKSDNSSHPNKDEPEYLPRETIVSSGIITSSS-
CIGSSA_084099.t1                    IIG-AEFSRSKSDNSSHPNKDEPEYLPREAIVSSGNITSSS-
Om_S1P1_CDQ90261.1_Scaffold_60      IMG-AEFSRSKSDNSSHPNKDEPEYSPRETVVSSGNITSSS-
Ssalar_S1P1                         IMG-AEFSRSKSDNSSHPNKDEPEYSPRETVVSSGNITSSS-
Drerio_S1P1                         IMG-AEFSRSKSDNSSHPNKDEPEYSPRETIVSSGNITSSS-
Oniloticus_S1P1                     IMG-AEFSRSKSDNSSHPNKEEVEYSPRETTVVSSGNVTSSS
Mmusculus_S1P1                      IIPGMEFSRSKSDNSSHPQKDDGDNP--ETIMSSGNVNSSS-
Rnorvegicus_S1P1                    IIPGMEFSRSKSDNSSHPQKDDGDNP--ETIMSSGNVNSSS-
Hsapiens_S1P1                       IIAGMEFSRSKSDNSSHPQKDEGDNP--ETIMSSGNVNSSS-
Ggallus_S1P1                        IIGGMEFSRSKSDNSSHPQKEEGDRP--ETIMSSGNVTSSS-
Xtropicalis_S1P1                    IITGMEFSRSKSDNSSHPQKDEGEYP--VTLMSSGNVTSSS-
                                    *:   *************:*:: :     : : *.   :**
```

Supplementary Fig. S2: Percentage amino acid identity and similarity among vertebrate S1P1. Accession numbers used were: Hs S1P1 (AAH18650.1 Sphingosine-1-phosphate receptor 1 [Homo sapiens]); Hs S1P2 (AAH69598.1 Sphingosine-1-phosphate receptor 2 [Homo sapiens]); Mm S1P1 (AAH51023.1 Sphingosine-1-phosphate receptor 1 [Mus musculus]); Rn S1P1 (NP_058997.1 Sphingosine 1-phosphate receptor 1 [Rattus norvegicus]); Gg S1P1 (XP_422305.3 PREDICTED: sphingosine 1-phosphate receptor 1 [Gallus gallus]); Xt S1P1 (NP_001072893.1 sphingosine 1-phosphate receptor 1 [Xenopus tropicalis]); On S1P1 (XP_005475711.1 PREDICTED: sphingosine 1-phosphate receptor 1 [Oreochromis niloticus]); Dr S1P1 (AAG45430.1 sphingosine 1-phosphate receptor [Danio rerio]); Ss S1P1 (XP_014071480.1 PREDICTED: sphingosine 1-phosphate receptor 1 [Salmo salar]); Ss S1P1-like (CIGSSA_084099.t1); Om S1P1 (CDQ90261.1 and Scaffold 6065) and Om S1P1 (CDQ69631.1).

	1	2	3	4	5	6	7	8	9	10	11
1. Om_S1P1_CDQ90261.1		97	67.8	67.2	89.2	87.5	99.7	67.6	65.9	67.8	97.6
2. Om_S1P1_CDQ69631.1	98.9		67.8	67	88.4	86.2	96.8	67.6	65.9	67.9	98.1
3. Mm_S1P1	81.4	81.2		94.2	68.4	66.5	67.9	98.4	73.3	84.8	67.5
4. Hs_S1P1	82.5	82.2	97.1		67.3	65.5	67.2	93.2	73.7	84.3	66.9
5. Dr_S1P1	93.3	92.2	81.7	81.4		84.3	89.2	68.2	66.5	68.3	88.2
6. On_S1P1	92.6	91.8	81.4	81.4	91		87.5	65.3	65.7	67.1	86.7
7. Ss_S1P1	99.7	98.7	81.4	82.5	93.3	92.3		67.8	65.6	67.9	97.3
8. Rn_S1P1	81.5	81.2	99	96.6	81.7	80.9	81.5		72.8	84.6	67.3
9. Xt_S1P1	80.7	81	84.8	85.6	79.4	80.9	80.5	84.3		73.1	65.6
10. Gg_S1P1	81	81.5	90.1	90.3	80.7	80.7	81.8	90.1	83.9		67.8
11. Ss_S1P1_LIKE	99.2	99.2	80.9	81.9	92.5	92.3	98.9	80.9	80.7	81	

identity
similarity

Supplementary Fig. S3: Neighbour Joining Tree of vertebrate S1P1. Phylogenetic tree was constructed using MEGA6 with 10,000 bootsrap value. Human S1P1 was used as a tree outlier. Accession numbers used were: Hs S1P1 (AAH18650.1 Sphingosine-1-phosphate receptor 1 [Homo sapiens]); Hs S1P2 (AAH69598.1 Sphingosine-1-phosphate receptor 2 [Homo sapiens]); Mm S1P1 (AAH51023.1 Sphingosine-1-phosphate receptor 1 [Mus musculus]); Rn S1P1 (NP_058997.1 Sphingosine 1-phosphate receptor 1 [Rattus norvegicus]); Gg S1P1 (XP_422305.3 PREDICTED: sphingosine 1-phosphate receptor 1 [Gallus gallus]); Xt S1P1 (NP_001072893.1 sphingosine 1-phosphate receptor 1 [Xenopus tropicalis]); On S1P1 (XP_005475711.1 PREDICTED: sphingosine 1-phosphate receptor 1 [Oreochromis niloticus]); Dr S1P1 (AAG45430.1 sphingosine 1-phosphate receptor [Danio rerio]); Ss S1P1 (XP_014071480.1 PREDICTED: sphingosine 1-phosphate receptor 1 [Salmo salar]); Ss S1P1-like (CIGSSA_084099.t1); Om S1P1 (CDQ90261.1 and Scaffold 6065) and Om S1P1 (CDQ69631.1).

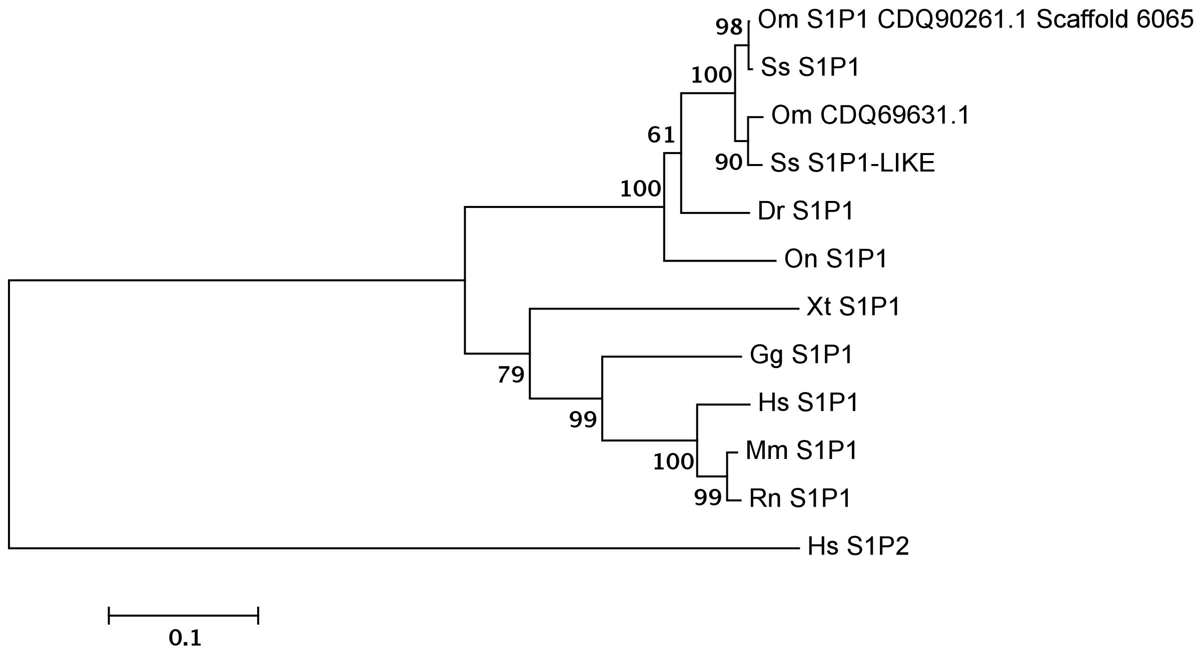

Supplementary Fig. S4: Conserved synteny of vertebrate S1P1 genes generated by Genomicus. Rainbow trout scaffold 6065 is not shown due to lack of the complete annotation of the scaffold. The central vertical line indicates the position of S1P1. From that line moving left, we found DPH5 homolog (*S. cerevisiae*) [Source:ZFIN;Acc:ZDB-GENE-041114-85], solute carrier family 30 (zinc transporter), member 7 [Source:ZFIN;Acc:ZDB-GENE-030131-5650], vascular cell adhesion molecule 1 [Source:ZFIN;Acc:ZDB-GENE-070209-238], G protein-coupled receptor 88 [Source:HGNC Symbol;Acc:4539], CDC14 cell division cycle 14 homolog A, b [Source:ZFIN;Acc:ZDB-GENE-070705-309], RNA terminal phosphate cyclase domain 1 [Source:ZFIN;Acc:ZDB-GENE-030131-9687], dihydrolipoamide branched chain transacylase E2 [Source:ZFIN;Acc:ZDB-GENE-050320-85], leucine rich repeat containing 39 [Source:ZFIN;Acc:ZDB-GENE-050417-279] and coiled-coil domain containing 76 [Source:ZFIN;Acc:ZDB-GENE-050327-19].

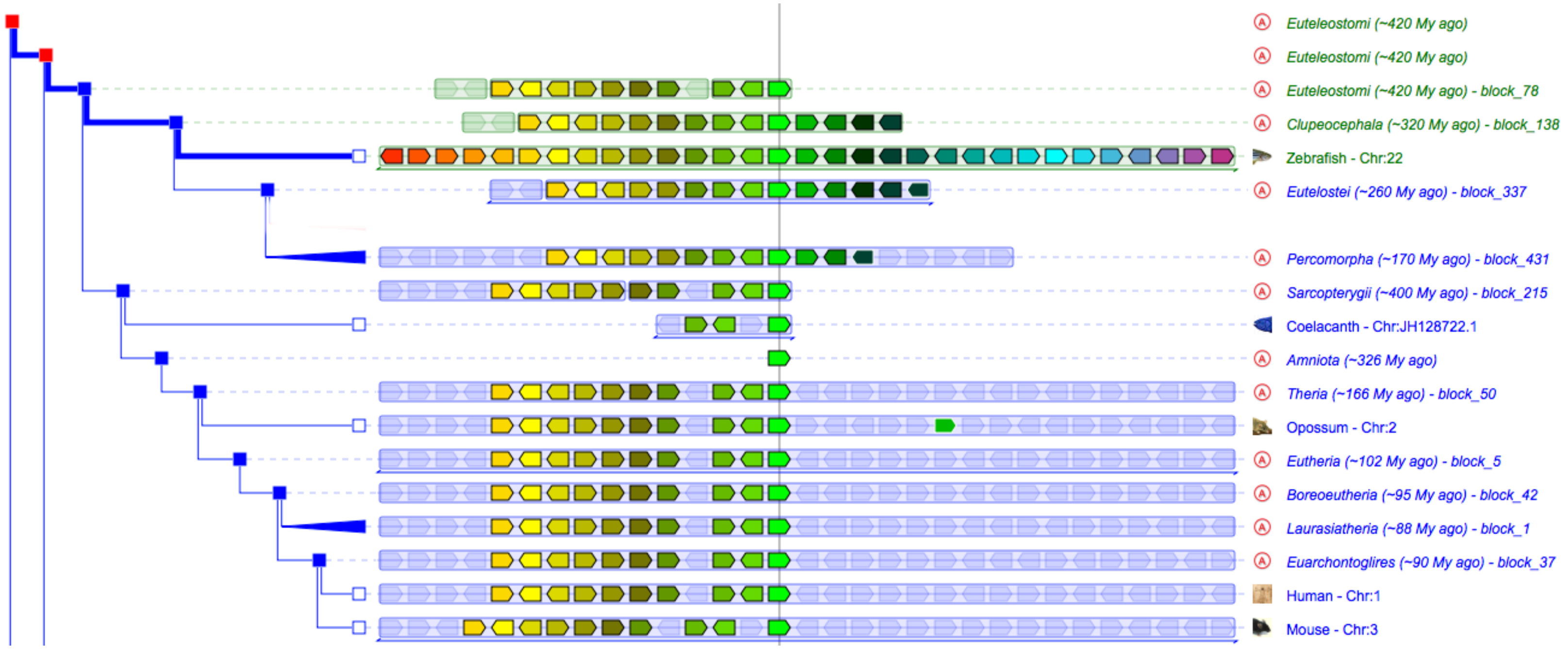

Supplementary Fig. S5: Immunoblots detecting IgM or IgT in supernatants obtained from rainbow trout HK and gill explants incubated for 5 days with control DMEM medium (C), 10^4 cfu/ml *F. major* (F), BSA (B) or *F. major* sphingolipids conjugated with BSA (S). Relative IgM and IgT levels were quantified by densitometry. Images show three different fish samples that are representative of two independent experiments with N=5 in each experiment.

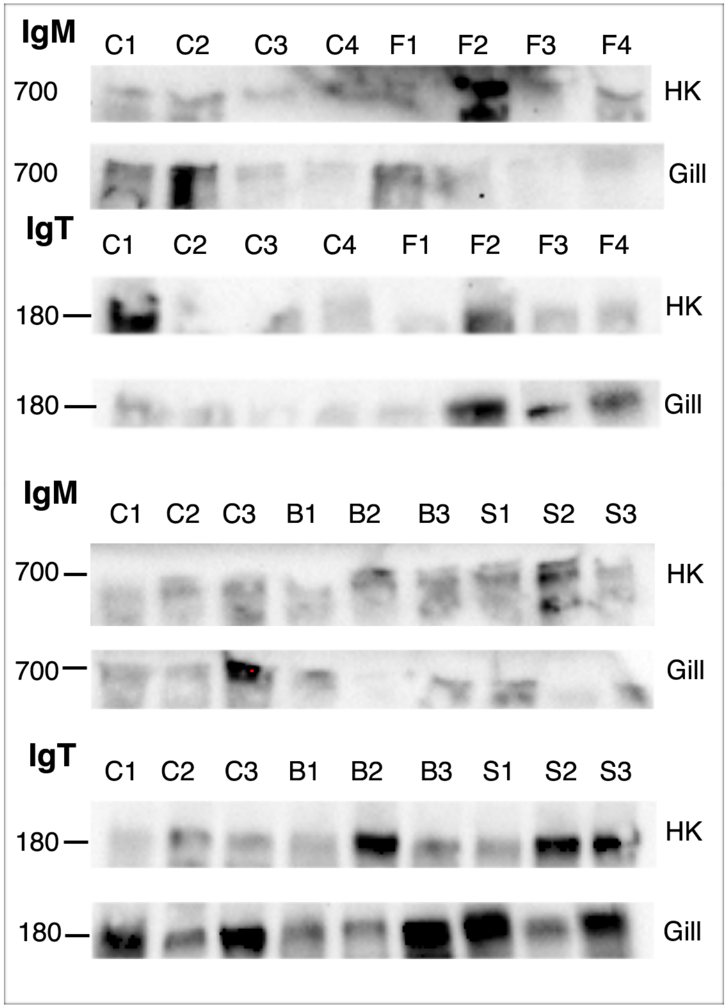

Supplement: Supplementary Information [file srep39054-s1.pdf]
